# Why Is Aging a Risk Factor for Cognitive Impairment in Parkinson's Disease?—A Resting State fMRI Study

**DOI:** 10.3389/fneur.2019.00267

**Published:** 2019-03-22

**Authors:** Atsuko Nagano-Saito, Pierre Bellec, Alexandru Hanganu, Stevan Jobert, Béatriz Mejia-Constain, Clotilde Degroot, Anne-Louise Lafontaine, Jennifer I. Lissemore, Kelly Smart, Chawki Benkelfat, Oury Monchi

**Affiliations:** ^1^Centre de Recherche, Institut Universitaire de Gériatrie de Montréal, Montreal, QC, Canada; ^2^Department of Neurology & Neurosurgery, and Psychiatry, McGill University, Montreal, QC, Canada; ^3^Université de Montréal, Montreal, QC, Canada; ^4^Cumming School of Medicine, Hotchkiss Brain Institute, Calgary, AB, Canada; ^5^Department of Clinical Neurosciences and Department of Radiology, University of Calgary, Calgary, AB, Canada; ^6^Movement Disorders Unit, McGill University Health Center, Montreal, QC, Canada; ^7^Department of Neurology, Montreal Neurological Hospital, Montreal, QC, Canada; ^8^Centre Hospitalier de l'Université de Montréal, Montreal, QC, Canada

**Keywords:** Parkinson's disease, mild cognitive impairment, age, functional connectivity, cognition, neuroimaging (functional)

## Abstract

Using resting-state functional MRI (rsfMRI) data of younger and older healthy volunteers and patients with Parkinson's disease (PD) with and without mild cognitive impairment (MCI) and applying two different analytic approaches, we investigated the effects of age, pathology, and cognition on brain connectivity. When comparing rsfMRI connectivity strength of PD patients and older healthy volunteers, reduction between multiple brain regions in PD patients with MCI (PD-MCI) compared with PD patients without MCI (PD-non-MCI) was observed. This group difference was not affected by the number and location of clusters but was reduced when age was included as a covariate. Next, we applied a graph-theory method with a cost-threshold approach to the rsfMRI data from patients with PD with and without MCI as well as groups of younger and older healthy volunteers. We observed decreased hub function (measured by degree and betweenness centrality) mainly in the medial prefrontal cortex (mPFC) in older healthy volunteers compared with younger healthy volunteers. We also found increased hub function in the posterior medial structure (precuneus and the cingulate cortex) in PD-non-MCI patients compared with older healthy volunteers and PD-MCI patients. Hub function in these posterior medial structures was positively correlated with cognitive function in all PD patients. Together these data suggest that overlapping patterns of hub modifications could mediate the effect of age as a risk factor for cognitive decline in PD, including age-related reduction of hub function in the mPFC, and recruitment availability of the posterior medial structure, possibly to compensate for impaired basal ganglia function.

## Introduction

In Parkinson's disease (PD), cognitive deficits are frequently present even in the early course of disease development (1). It has been reported that up to 40% of patients with PD have mild cognitive impairment (MCI) (2). Furthermore, patients with PD and MCI have a higher risk of developing dementia compared with patients who do not have MCI (3). In patients with MCI, deficits are neither severe enough to interfere considerably with daily life nor reach criteria for dementia (recent guidelines for MCI diagnosis in PD) (4), but the early presence of MCI in PD has a significant effect on the incidence of dementia at later stages of PD (5, 6). Aging is a risk factor for MCI in PD. Having MCI in PD was associated with older age at assessment and at disease onset (7). Aging is also the strongest predictive factor of dementia in PD patients (2). Untangling the effect of the neuro-degeneration of PD from the effects of regular aging is important for further understanding the functional connectivity in PD patients associated with cognitive deficits. A recent study support this, showing a strong effect of aging on PD patients' cognition (8).

Various studies related to resting-state functional MRI (rs-fMRI) involving PD patients have been published to date (9–11), but discrepancies in brain connectivity findings have been frequently observed. For example, in PD patients, both decreased (12–14) and increased (12, 15–17) cortico-striatal connectivity have been observed. The discrepancy could be related to clinical differences in the patient samples (e.g., cognitive, emotional, motor dysfunctional states, but also age). However, methodological differences might also induce discrepancies. Moreover, even when using the same approach (e.g., comparison of the connectivity strength between brain regions), selection of the brain regions (location and number) could affect the results (18).

The application of graph theory methods to brain imaging data is a simple and powerful mathematical framework for the characterization of topological features of brain networks (19, 20). Intrinsic patterns of functional connectivity in the human have been established, such as in the visual, auditory, somatosensory-motor, task-control, attention, and default mode networks (21–23). When the brain network is designed to be calculated on binary graphs with graph theory approaches, highly connected regions in networks with other regions in the brain are defined as hubs, including the “provincial hubs” (indicating more local connectivity) and “connector hubs” (indicating long range connectivity between different brain networks). A previous study with younger people using graph theory revealed that each intrinsic network (or module) connects each other via specific connector hubs, including medial structures such as the medial prefrontal cortex (mPFC), anterior and posterior cingulate cortex, and medial posterior parietal cortex (24–26). The connectivity between the anterior and posterior medial cortices, and connector hub function of the prefrontal cortex is lowered in older people (27, 28).

The basal ganglia is known to receive many connections from most of the cortex (29), and considered to function by integrating different modules (30, 31). Therefore, one might think that the basal ganglia can act as connector hubs, which have many numbers of the connectivity during resting-state. However, in a previous analysis the basal ganglia did not emerge as a hub in both younger and older healthy individuals, with only limited number of connectivity with other brain regions (28), although this region acts as a “module connector,” supporting connectivity between different networks. Our previous studies indicated that the cortico-striatal connectivity increased significantly while performing a cognitive task (32), plausibly acting to link separate networks in a task-dependent manner (33). The nigrostriatal dopaminergic pathology in PD patients (34) could result in impairments of this network-integration function (33). In our longitudinal study with PD patients, increased activation in medial cortical structures (e.g., the mPFC and the precuneus) during a cognitive task was observed both in PD-non-MCI and PD-MCI when they performed an executive task at the second time, compared to the first time point (35). One possibility is that these medial regions are recruited in PD patients to compensate for the loss of “module connector” function of the basal ganglia seen in healthy people.

Here, we aimed to investigate the connectivity change PD patients associated with MCI and aging, in two steps. In the first step, we analyzed rs-fMRI data of three groups, Older healthy volunteers (OHV), PD-non-MCI, and PD-MCI, which were collected with the same scanning protocol. The bootstrap analysis of stable cluster (BASC) method (36) was applied in a data-driven way, to see the impact of selection of “*clusters,”* in the brain. Then, the connectivity strength of each cluster between groups were compared. This allowed to compare the effect of clusters selection at different resolutions and locations. In a second step, using a set of clusters from step 1, we applied a graph-theory approach to data from four groups of interest: PD-non-MCI, PD-MCI, OHV, and young healthy volunteers (YHV), and investigated differences in regional hub function in the brain. We applied the cost-threshold approach adjusting the number of connections in all the participants, rather than connectivity strength-threshold approach, because the former is more stable and relevant in the analysis of connectivity “patterns” between different populations (33, 37, 38). Together these analyses allowed us to explore the independent and overlapping relationships among aging, pathology, cognitive capability, and brain connectivity in PD. We predicted that the connectivity results would reflect the importance of age as an important factor for cognitive deficits in PD.

## Methods

### Subjects

Thirty-five non-demented PD patients at stages I and II of Hoehn and Yahr (mean age ± SD, 66.2 ± 7.6 years; range, 50–85; 20 male and 15 female patients) were recruited and subsequently divided into two groups: those with MCI (PD-MCI; *n* = 15) and those cognitively intact (PD-non-MCI, *n* = 20). The sample size was determined based on our previous study of functional MRI, comparing the PD-non-MCI vs. PD-MCI (19 vs. 14) (39). Inclusion criteria for MCI were based on the Movement Disorder Society Task Force guideline for PD (6), based on five cognitive domains (Table 1). Objective evidence of cognitive decline defined as performance (1) standard deviation below the standardized mean (taking into account age and sex) in at least two measures within the same cognitive domain of the neuropsychological assessment. (2) Subjective complaint about cognitive decline by the patient or accompanying person; (3) Absence of significant decline in daily living activities; and (4) Absence of dementia. PD patients underwent cognitive assessment and fMRI and took their usual level of dopaminergic medication during these sessions. As a control group, 21 non-MCI older volunteers (mean age ± SD, 70.0. ± 5.4 years; range, 62–78; 5 male and 16 female patients) were recruited, and also underwent cognitive assessment and MRI within 2 weeks of each other. The same criteria were used in the control group, including a neuropsychological assessment to exclude the presence of MCI.

**Table 1:** Cognitive assessment.

**Cognitive domain**	**Test**	**References**
Attention	Digit Span	Wechsler, 1997
	Digit Symbol	Wechsler, 1997
Executive	Stroop	Golden and Freshwater, 1998
	Trial Making Test B, Time, and Error	Reitan and Wolfson, 1985
	Brixton	Burgess and Shallice, 1997
	Montreal d'Evaluation de la communication (MEC), Verbal fluency-orthographc criteria subtest	Joanette et al., 2004
Memory	Rey-Osterrieth Figure copy	Osterrieth, 1944
	Rey Auditory Verbal Learning Test (RAVLT)	Schmidt, 1996
	Logical Memory subtest of Wechsler Memory Scale (WMS-III)	Wechsler, 1999
Visuospatial	Hooper test	Hooper, 1958
	Rey-Osterrieth figure copy	Osterrieth, 1944
	Montreal Cognitive Assessment (MoCA), Clock drawing	Nasreddine et al., 2005
Language	MEC, semantic subtest	Joanette et al., 2004
	Boston Naming Test	Kaplan et al., 1983

*References for cognitive tasks are shown in the Supplementary Information*.

Demographic details are given in Table 2. A significant difference in age occurred between the PD-non-MCI and PD-MCI (Table 2). Matching for age of PD-non MCI and PD-MCI, however, might induce a recruitment bias, as many studies indicate that PD patients with MCI are generally older than PD-non MCI (2, 7, 8). Here, we opted for another strategy to investigate the effect of age, we added a young healthy group allowing us to investigate of age vs. the effect of disease. Of note, UPDRS scores for six PD patients (PD-non-MCI; 3, PD-MCI; 3) were missing.

**Table 2:** Demography of participants.

**Group**	**OHV**	**PD-non-MCI**	**PD-MCI**	
Number	21	20	15	
Age	70.0 ± 5.4 (62–78)	63.8 ± 7.4 (50–78)	69.4 ± 6.8 (61–85)	*,***
Sex(M:F)	5:16	10:10	10:5	*, **
Disease duration		6.7 ± 3.5	9.3 ± 5.2	
UPDRS (motor score)		26.7± 12.1 (*n* = 17)	29.9 ± 11.7 (*n* = 12)	
Education	15.5 ± 3.2	15.0 ± 2.2	14.8 ± 2.5	
MoCA	28.2 ± 1.5	28.5 ± 1.6	26.9 ± 2.0	**, ***
BDI-II	4.3 ± 4.2	7.7 ± 5.6	10.1 ± 4.9	*, **
L-DOPA equivalent dosage		496 ± 428	531 ± 394	

*t-test or χ^2^-test*.

* *p < 0.05 in HV vs. PD-non-MCI*.

** *p < 0.05 in HV vs. PD-MCI*.

*** *p < 0.05 in PD-non-MCI vs. PD-MCI*.

rsfMRI data from 30 young participants (mean age ± SD, 23.8 ± 3.17 years; range, 20–30; 14 male and 16 female) were obtained. The data was collected for two other studies [Transcranial magnetic stimulation, *n* = 16, (not published), and positron emission tomography (PET), *n* = 14 (40)].

### Resting-State fMRI Acquisition

All participants were scanned with 3T Siemens TIM MRI scanners at the Institut Universitaire de Gériatrie de Montréal (OHV, PD-non-MCI, PD-MCI, and YHV-TBS), and at the McConnell Brain Imaging Center, Montreal Neurological Institute, McGill University (YHV-PET). Sessions began with high-resolution, T1-weighted, 3D volume acquisition for anatomic localization (voxel size 1 mm^3^), followed by “resting-state” echo-planar T2^*^-weighted image acquisitions with BOLD contrast. The parameters for echo-planar T2^*^-weighted images were different; for OHV, PD-non-MCI, and PD-MCI, TR = 2.6 s, echo time, 30 ms; flip angle, 90°, volume number 150, slice number, 42, matrix size, 64 × 64 pixels; voxel size, 3.4 × 3.4 × 3.4 mm^3^; for YHV in TBS study, TR = 2.5 s, echo time, 30 ms; flip angle, 90°, 252 volumes, slice number, 41, matrix size, 64 × 64 pixels; voxel size, 3.5 × 3.5 × 3.5 mm^3^; and for YHV in PET study, TR = 2.11 s, echo time, 30 ms; flip angle, 90°, 180 volumes, slice number, 40, matrix size, 64 × 64 pixels; voxel size, 3.5 × 3.5 × 3.5 mm^3^. The OHV and PD participants had three runs of T2^*^-weighted image acquisitions. All scans were used for the first step analysis of comparison of the connectivity strength among the OHV, PD-non-MCI, and PD-MCI groups, but only the first run was used in order to make comparisons with the YHV (who were scanned only once) for the second step analysis using the graph theory approach. During rsfMRI, OHV, PD-non-MCI, PD-MCI, and YHV-TBS groups were presented with a white screen and a black cross in the middle, and YHV-PET participants were presented with a white screen. They were instructed to keep their eyes open (to avoid falling asleep), focus on the cross or white screen, and relax.

Of note, we confirmed that there were no significant differences in network properties (e.g., global and local efficiency, degrees and betweenness centrality of all the clusters, using the methods described below in Step 2) between the two sets of participants, even without correction for multiple comparisons. Therefore, we combined the two data set into one YHV group for this study. All the participants provided informed consent, and the protocol was approved by the Research Ethics Committee of the Regroupement Neuroimagerie Québec.

#### rsfMRI Data Processing

All the data were pre-processed with the same procedure. We applied the NIAK pre-processing pipeline to the fMRI datasets (41). First, slice timing correction was performed with spline interpolation. After motion correction, slow time drift was removed from the blood oxygen level-dependent (BOLD) time series with a high-pass filter of 0.01 Hz. To avoid possible artificial correlation induced by low-pass filters (42), no low-pass filter was applied. To minimize artifacts due to excessive motion, all time frames showing a displacement > 0.5 mm were removed. A minimum of 40 un-scrubbed volumes per run was required for further analysis, and no scans were removed based on this criterion except the third run of one participant belonging to the PD-non-MCI group. The mean motion-corrected time-averaged functional volume was co-registered with the individual T1 scan (43), then transformed into the ICBM152 space using the acquired parameter at a 3 mm isotropic resolution. The following nuisance covariates were regressed out from fMRI time series: slow time drifts (basis of discrete cosines with a 0.01 Hz high-pass cut-off), average signals in conservative masks of the white matter and the lateral ventricles, and the first 3–10 principal components of the six rigid-body motion parameters and their squares (44, 45). The fMRI volumes were finally spatially smoothed with a 6 mm isotropic Gaussian blurring kernel.

### Step 1. Connectivity Strength Between Clusters in OHV, PD-non-MCI, and PC-MCI

#### Bootstrap Analysis of Stable Clusters (BASC)

We applied the BASC algorithm to identify clusters that consistently exhibited similar spontaneous BOLD fluctuations in individual subjects and were spatially stable across subjects (46). We first applied a region-growing algorithm to reduce each fMRI dataset into a time × space array, with 957 regions (47). BASC replicates a hierarchical Ward clustering 1,000 times and computes the probability that a pair of regions fall in the same cluster, a measure called stability (46). The region × region stability matrix is fed into a clustering procedure to derive consensus clusters, which are composed of regions with a high average probability of being assigned to the same cluster across all replications. At the individual level, the clustering was applied to the similarity of regional time series, which was replicated using a circular block bootstrap. Consensus clustering was applied to the average individual stability matrix to identify group clusters. The group clustering was replicated via bootstrapping of subjects in the group. A consensus clustering was finally applied on the group stability matrix to generate group consensus clusters. The cluster procedure was carried out at a specific number of clusters (having a corresponding “resolution”). Using a “multiscale stepwise selection” (MSTEPS) method (48), we determined a subset of resolutions that provided an accurate summary of the group stability matrices generated over a fine grid of resolutions: K = [4, 10, 19, 35, 63, 118, 221, 393].

#### Derivation of Functional Connectomes

For each resolution K, and each pair of distinct clusters, the between-clusters connectivity was measured by the Fisher transform of the Pearson's correlation between the average time series of the clusters. The within-cluster connectivity was the Fisher transform of the average correlation between time series inside the cluster. An individual connectome was thus a K × K matrix.

#### Statistical Testing

To test for differences between, OHV vs. PD-non-MCI, OHV vs. PD-MCI, and PD-non-MCI vs. PD-MCI at a given resolution, a general linear model (GLM) for each connection between two clusters was applied. A GLM included an intercept, and the average frame displacement of the runs involved in this analysis (without including age as a covariate). In addition, a GLM including age as a covariate was also calculated to see the impact of age on the comparisons. The contrasts of interest (HV vs. PD-non-MCI, HV vs. PD-MCI, and PD-non-MCI vs. PD-MCI) was represented by a dummy covariate coding the difference in average connectivity between the two groups.

The false-discovery rate (FDR) across connections was controlled at qFDR ≤0.05 (49). We assessed the impact of that parameter by replicating the GLM analysis at the eight resolutions selected by MSTEPS. We implemented an omnibus test (family-wise error rate α ≤ 0.05) to assess the overall presence of significant differences between groups, pooling FDR results across all resolutions (18). If the omnibus test across resolutions was not significant, then no test would be deemed significant. Since this omnibus test was significant, we used the FDR threshold of *q* ≤ 0.05 to explore single resolutions.

### Step 2. Graph Theory Methodology

To investigate the between-network functional connectivity of the brain using a cluster-based method, a graph theory approach was applied (50). The same set of functional clusters (*n* = 118) as in step one was used. This set was selected because it included the maximal number of the clusters which showed significant differences between the PD-non-MCI and PD-MCI (see Results). The time series of the BOLD signal of each cluster, which was considered as a node in graph theory approach, were extracted for each participant. Then, the Pearson correlations were calculated between each pair of the 118 clusters, resulting in a symmetric 118 × 118 correlation matrix. We then applied a cost-threshold approach to the correlation matrix, as in our previous studies (33, 37). The cost is defined as the ratio between the actual number of connections and the maximum number of possible connections between every two clusters. As a function of cost, network features, such as global, local, cost efficiencies, were calculated. Global efficiency is an index of inverse path length, defined by an average minimum number of connections that link any two nodes of the network, and indicates the efficiency of information transfer among different brain regions (50). The cost efficiency is defined as “global efficiency—cost,” and it is assumed that the brain operates optimally with the maximum cost efficiency, maximizing information transfer (24, 50). As a function of the cost between 0.5 and 50% with 0.5% step, cost efficiency was calculated to examine the economical cost, while maximizing cost efficiency. This process was identical to our previous study (33). We also confirmed the small-worldness with a parameter “omega,” which is typically near 0 for networks with small-world properties (51). The average cost efficiency in each group was maximized which resulted in a range between 19.5 and 22%, depending on the group (YHV, 19.5%; OHV, 22.0%, PD-non-MCI, 20.0%; PD-MCI, 21.5%). Therefore, we used the cost (18–24%) with 0.5% step, where the omega was between −0.03 and 0.07 in all the groups, to generate whole brain networks with the 118 clusters. With each of the 13 costs, network features (degree, and between centrality, see below) were calculated and averaged individually, for further analysis. Graphs of the “cost efficiency” and omega, as the function of the cost are shown in Figure S1.

**Figure 1:**
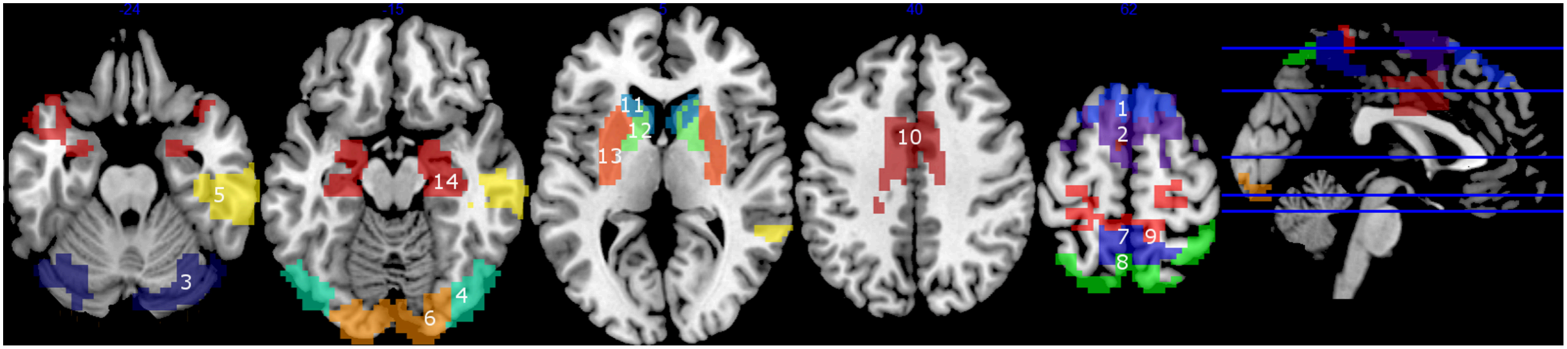
Clusters with high degrees (> mean + 1 S.D.) (1–10), and the clusters in the basal ganglia and the hippocampus (11–14). 1, mPFC1; 2, mPFC2; 3, Cerebellum; 4, Middle Temporal; 5, right Temporal; 6, Occipital; 7, Precuneus1; 8, Precuneus2; 9, Precuneus+Primary Motor Area; 10, Cingulate Gyrus; 11, Caudate; 12, Globus pallidus; 13, Putamen; 14, Hippocampus.

In graph theory analysis, degree is the number of connections attached to a given node in a designed binary graphed brain network, and betweenness centrality is the total number of all shortest paths linking to the given node (52). Hubs are nodes with high degree, or high centrality (19). For each cluster, in each participant, first, the degrees were calculated. We selected all the clusters which indicated more than mean + 1S.D. of all the clusters of all the subjects, at least in one group, considering them as hubs (53). Using these selected clusters, the betweenness centrality was also calculated. This was added based on the hypothesis that if the hub regions function effectively for the information transfer in the brain network, the nodes also have high betweenness centrality. In addition to the hub clusters, we examined clusters located in the basal ganglia (caudate, putamen, and globus pallidus) and hippocampus, because they are considered as important regions for integrating information from different brain modules (30, 54), and we have observed that they are the key regions of cognitive decline in PD patients (39, 55, 56). The total cluster number was 14 (Figure 1).

#### Group Difference of Degree and Betweenness Centrality

To examine the impact of pathology, age, and cognition separately, group difference (YHV vs. OHV, OHV vs. PD-non-MCI, PD-non-MCI vs. PD-MCI) of the degree and the betweenness centrality were investigated with student *t*-test in each hub-cluster. For the comparisons of OHV vs. PD-non-MCI and PD-non-MCI vs. PD-MCI, analyses including age as a covariate, were also performed applying a general linear model. Multiple comparisons with the selected clusters (*n* = 14) was applied and a significance threshold was determined at pFDR < 0.05. Predicted regions which have been linked to PD pathology and/or cognitive deficits in PD (e.g., caudate, globus pallidus, putamen, hippocampus, mPFC, posterior cingulate cortex, and precuneus) were also reported without multiple comparison correction using a threshold of *p* < 0.05.

#### Demographic Impact on Group Difference of Degree in Precuneus and Cingulate Cortex

Studies indicate that cognition in PD patients is associated not only with age, but also gender, depression, education, and severity of motor symptoms (7, 8). Accordingly, there also existed group difference between the PD-non-MCI and PD-MCI, in sex, and Beck's Depression Inventory II (BDI-II, as an index of depression), but not in education or UPDRS motor scores. It should be noted that UPDRS data were missing in six participants. We also performed the analyses described below on the 29 patients and found similar results as with the full 35 PD patients. Here, we only report the results with the full PD group.

Among the observations in the above section of “*Group difference of degree and betweenness centrality,”* we were especially interested in the clusters (the precuneus and the cingulate cortex), which showed increased degrees in the PD-non-MCI compared to the OHV. To see the impacts of age, sex, and BDI-II, on the degrees in the clusters, we further performed group comparisons (OHV, PD-non-MCI, and PD-MCI), dividing each group into two subgroups according to age (younger vs. older), sex (male vs. female), and BDI-II (lower vs. higher), separately. Thresholds for dividing a group were set at 67.6 for the age, and 6.9 for the BDI-II. They were determined at the average of the whole groups (see **Table 4**). The average of the degree of the precuneus and the cingulate cortex (Figure 1, # 7, 8, 9 10) was calculated for each participant. Then the averaged degree was compared with two types of two-way ANOVA (group × subgroup), and two types of *t*-tests. One type of the ANOVA was with three groups (OHV, PD-non-MCI, and PD-MCI) × two subgroups of age, sex, and BDI, separately. The other type was with two groups (OHV and PD-non-MCI) × two subgroups of age, sex, and BDI-II, separately. Simple *t*-tests of OHV vs. PD-non-MCI, and PD-non-MCI vs. PD-MCI, were performed in each subgroup.

Significant threshold was set at *p* < 0.05.

#### Correlation Between Degree and Cognitive Function

We hypothesized that the regions with significant difference in the group comparisons above are associated with cognitive performance. Therefore, correlation analysis between the degree and/or betweenness centrality of the regions, with the mean Z-scores of the five cognitive domains (attention, executive, memory, visuospatial, and language; see Table 1 for detail), was performed, in OHV and PD groups, separately. For the OHV, four regions (clusters # 1, 2, 12, 14, corresponding to the mPFC1 and 2, globus pallidus, and hippocampus) were selected based on group differences from the 14 clusters. For all PD patients (collapsing the PD-non-MCI and PD-MCI together), four regions (clusters # 7, 8, 9, 10, corresponding the three precuneus regions and the cingulate gyrus) were selected based on group differences from the 14 clusters. Multiple comparison with of all the correlations (*n* = 4) was applied at a significant threshold of pFDR < 0.05 in each group. Analyses with age covaried out was also performed for the PD patients, applying a general linear model.

## Results

### Cognitive Assessment

Among the 35 PD participants, 15 were grouped in PD-MCI. Eleven showed single domain cognitive impairment (attention: 2, executive: 6, memory: 0, visuospatial: 3, and language: 1), and three showed impairment on multiple domains. Participant demographics are shown in Table 2. Group difference was observed in age, sex, MoCA and Beck's Depression Inventory II (BDI-II) (Table 2). A marginal group difference was observed in disease duration (*p* = 0.089). The mean Z-scores of each domain are shown in the Figure S2. Student *t*-tests indicated that all the Z-scores on these measures were lower in PD-MCI than HV (*p* < 0.05). The Z-scores in the PD-MCI were lower than PD-non-MCI in attention, executive language domains (*p* < 0.05), and marginally lower in memory domain (*p* = 0.055). Between the HV and the PD-non-MCI, significant difference was observed in the executive and language domains (*p* < 0.05).

### Connectivity Analyses

#### Step 1. Connectivity Strength Between Clusters in OHV, PD-non-MCI, and PC-MCI

Across the resolutions, the omnibus tests indicated significant difference between the HV and PD-MCI groups (with age as a covariate), and between PD-non-MCI and the PD-MCI groups (without controlling for age), both indicating decreased connectivity in PD-MCI. Details are in Table 3. The results are stable across resolutions in PD-non-MCI vs. PD-MCI (without age as a covariate), showing a tendency of gradually decreasing percent of significantly different connectivities as the resolution increased. No difference was observed in OHV vs. PD-non-MCI comparisons (with age covariate). The detail results are shown in the SI. Briefly, when comparing PD-non-MCI with PD-MCI (without age covariate), with resolution 19, group connectivity differences was observed in most of the brain except the temporal area and the upper cerebellum (Figure S3, top). Discovery rate, indicating the rate of connections with significant effects for each cluster, were prominent in the medial part of the cortex corresponding to the motor area. With resolution 118, similar, but weaker group differences were observed (Figure S3, middle). Between the OHV vs. PD-MCI (with age covariate), with resolution 118, significant differences in the discovery rates were observed in the medial frontal motor cortex, the posterior cingulate cortex, right anterior prefrontal cortex, and occipital area (Figure S3, bottom).

**Table 3:** Group differences in connectivity strength between clusters between groups.

**Cluster number**	**4**	**10**	**19**	**35**	**63**	**118**	**221**	**393**	***p*-value**
**WITHOUT AGE AS A COVARIATE**									
HV vs. PD-non-MCI	0	0	0	0	0	0	0	0	1
HV vs. PD-MCI	0	0	0	0	0.001512	0.001149	0.001228	0.000699	0.0761
PD-non-MCI vs. PD-MCI	0.4375	0.26	0.085873	0.088980	0.011590	0.007182	0	0	0.0032
**WITH AGE AS A COVARIATE**									
HV vs. PD-non-MCI	0	0	0	0	0	0	0	0	1
HV vs. PD-MCI	0.125	0	0	0	0.005039	0.000862	0.001515	0.001463	0.0267
PD-non-MCI vs. PD-MCI	0	0	0.002770	0	0	0	0	0	0.0841

*The numbers under each cluster number indicate the proportion of significantly different connectivity over all possible connections. The p-value is based on the omnibus test across all the resolutions*.

#### Step 2. Graph Theory Analyses

##### Hub regions

**Figure 2:**
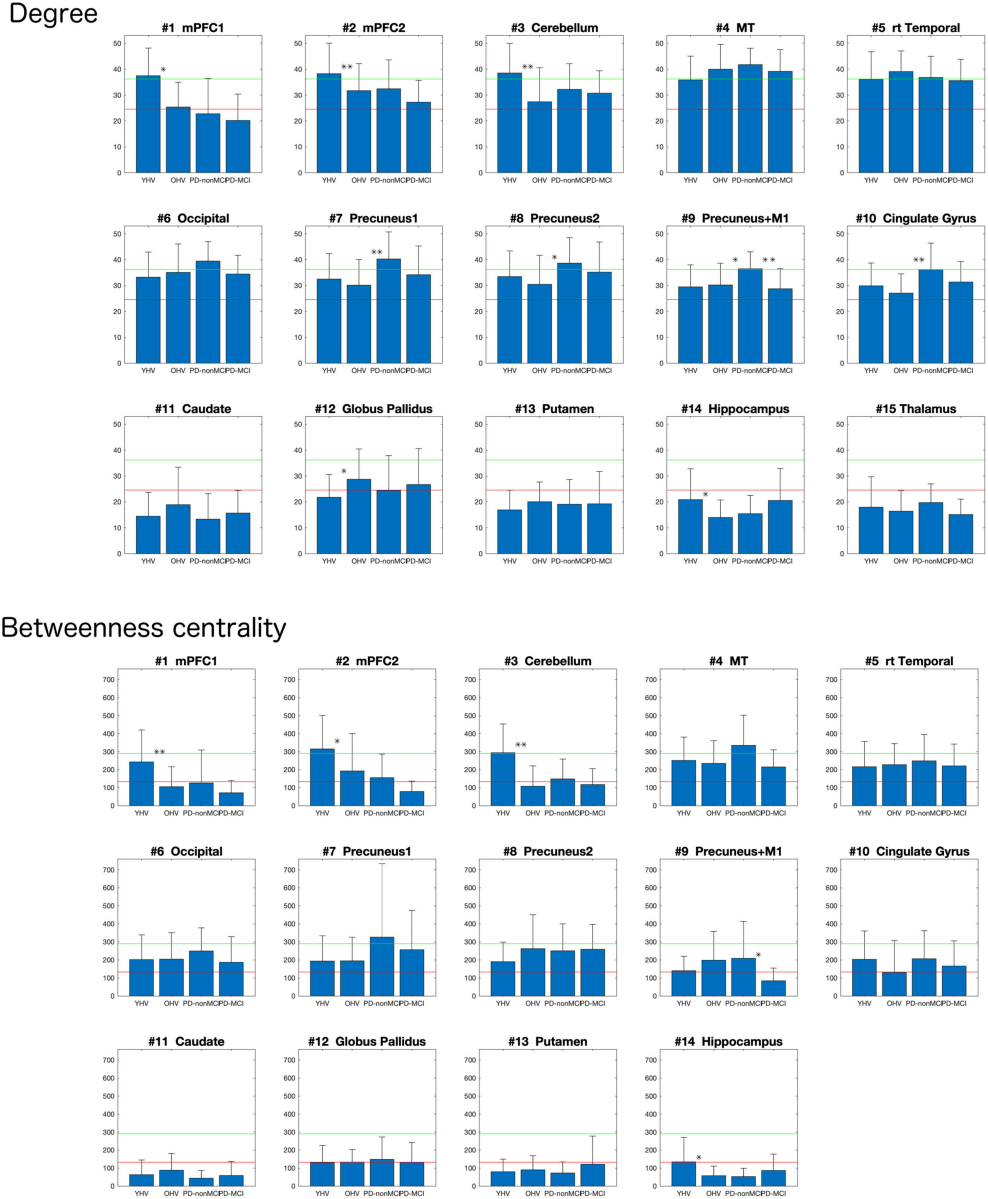
Mean degrees (top) and the betweenness centralities (bottom) of each cluster, depending to the groups (YHV, OHV, PD-non-MCI, and PD-MCI). **the significant difference with correction for multiple comparisons, and *the significant difference without multiple comparison. The bar indicates the S.D.

The average of the degrees and the betweenness centrality of all the 118 clusters of all the participants were 24.6 ±11.6 and 132.9 ± 11.6, respectively. Ten clusters showed degrees greater than mean + 1S.D in at least one group (Figures 1, 2, top). In the mPFC and the cerebellum (# 1, 2, 3) the higher (> mean + 1 S.D.) degrees were observed in YHV only. In four other medial structures covering the precuneus and cingulate gyrus (#7, 8, 9, 10) and in the occipital cortex (#6), the higher degrees were observed in PD-non-MCI, only. In the temporal and temporoparietal areas (#4,5), the higher degrees were observed in all the groups, except in PD-MCI for #5. Within these regions, higher betweenness centralities were observed in the mPFC and the cerebellum (#2, 3) in the YHV, and in the middle temporal area (#4) and the precuneus (#7), in PD-non-MCI (Figure 2, bottom).

##### Group difference of degree and betweenness centrality

Results are summarized in Figure 2.

*OHV vs. YHV*. The YHV indicated higher degrees in the mPFC and the cerebellum (#2, 3) compared with the OHV (adjusted *p*-values 0.0018 and 0.017, respectively). A similar decrease in OHV was observed in the other cluster of the mPFC (#1) (uncorrected *p* = 0.046, adjusted *p* = 0.13). In the *a priori* predicted regions, higher degrees were observed in the globus pallidus (#12) and lower degrees in the hippocampus (#14) in OHV compared to YHV, though this did not survive correction for multiple comparisons (uncorrected *p*-values 0.019 and 0.020, respectively; adjusted *p*-values 0.070 and 0.070). Higher betweenness centralities were also observed in the mPFC and in the cerebellum (#1 and #3) in YHV compared with OHV (adjusted *p*-values 0.019 and 0.0004, respectively). In the cluster of the mPFC (#2), reduction was also observed in OHV compared to YHV (uncorrected *p* = 0.0326; adjusted *p* = 0.114). Decreased betweenness centrality in the hippocampus was also observed in the OHV compared with YHV (uncorrected *p* = 0.018; adjusted *p* = 0.085).

*PD-non-MCI vs. OHV*. Higher degrees were seen in PD-non-MCI compared to OHV in the precuneus and the cingulate cortex (#7, 10; adjusted *p*-values 0.020 and 0.020, respectively). Increases in PD-non-MCI participants were also seen in the clusters of the precuneus regions (#8, 9; *p*-value without multiple comparison = 0.018 and 0.023, respectively, and adjusted *p*-value = 0.063 and 0.053, respectively). When age was included as a covariate, group difference between OHV and PD-non-MCI was observed in the precuneus and the cingulate cortex (#7, 8, 10; adjusted *p*-values 0.047, 0.047, and 0.047, respectively). In the other cluster of the precuneus (#9), the same pattern was observed (*p*-value without multiple comparison with *p* = 0.015 and adjusted *p* = 0.053). No difference was observed in the betweenness centrality, with or without age-covaried out (adjusted *p* > 0.3).

*PD-non-MCI vs. PD-MCI*. Higher degrees were seen in PD-non-MCI in the precuneus (#9; adjusted *p* = 0.046). No significant difference of degrees was observed between the PD-non-MCI and PD-MCI in the other clusters of the presumes (#7, 8,10) (*p*-value without multiple comparison > 0.1). Higher betweenness centrality in the presumes (#9) was observed in the PD-non-MCI group (*p* = 0.032 without multiple comparison; adjusted *p* = 0.20). Controlling for age, the same pattern was observed in the presumes (#9) (*p* = 0.044; adjusted *p* = 0.18). No other difference was observed in the betweenness centrality, with or without including age as a covariate (*p*-value without multiple comparison >0.50).

##### Demographic impact on group difference of degree in precuneus and cingulate cortex

All the results are shown in the Table 4. In ANOVA, strong group effects, but neither subgroup effects nor interactions were observed, with subgroups of age, sex, and BDI-II. However, marginal interaction was observed between the two groups (OHV vs. PD-non-MCI) and the age (< 67.6 vs. >67.6). In *t*-tests, in the younger participants (< 67.6) and in the female patients, the averaged degree was strongly higher in PD-non-MCI compared with OHV. The mean of the averaged degree of the clusters in each subgroup is shown in Figure 4.

**Table 4:** Group differences of averaged degrees in precuneus and cingulate cortex, between two subgroups of age, sex, and BDI.

		**Age**	**Sex**	**BDI**
Definition of subgroups	subgroup1	<67.6	Male	<6.9
	subgroup2	>67.6	Female	>6.9
**NUMBER**				
OHV	Subgroup1	8	5	17
	Subgroup2	13	16	4
PD-non-MCI	Subgroup1	12	10	9
	Subgroup2	8	10	11
PD-MCI	Subgroup1	7	10	4
	Subgroup2	8	5	11
**TWO-WAY ANOVA (*****p*****-value)**				
Group effect (OHV, PD-non-MCI, PD-MCI)		0.002	0.005	0.01
Subgroup effect (subgroup1, subgroup2)		0.705	0.261	0.344
Interuction (group × subgroup)		0.214	0.441	0.844
Group effect (OHV, PD-non-MCI)		<0.001	0.001	0.002
Subgroup effect (subgroup1, subgroup2)		0.943	0.829	0.259
Interuction (group × subgroup)		0.074	0.587	0.644
*t*-test (*p*-value)	Between groups			
OHV vs. PD-non-MCI	Subgroup1	0.001	0.083	0.015
	Subgroup2	0.119	0.002	0.055
PD-non-MCI vs. PD-MCI	Subgroup1	0.158	0.070	0.452
	Subgroup2	0.166	0.501	0.017

*Numbers of subgroups, p-values of two-way ANOVA and t-test are shown*.

##### Correlation between degrees and cognitive function

**Figure 3:**
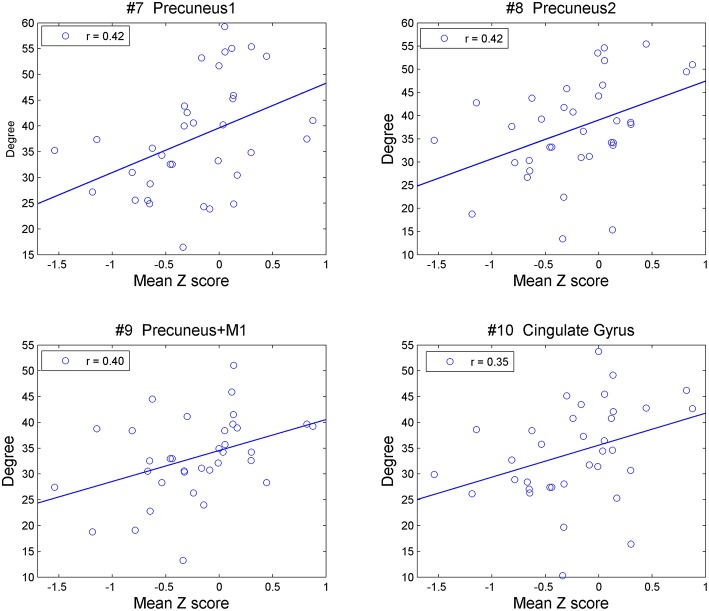
Correlation between degrees of the precuneus (#7, 8, 9) and cingulate cortex (#10) with mean Z-values over all five cognitive domains in all the PD patients.

In all PD patients, the degrees of the clusters of the presumes and the cingulate cortex (#7, 8, 9, 10) were positively correlated with the mean Z-score across all domains of the cognitive assessment (Figure 3). The correlation rates (*r*) were, 0.42, 0.42, 0.40, and 0.35, and the adjusted *p*-values were 0.027, 0.027, 0.27, and 0.042, respectively. When age was included as a covariate, significant correlations were observed in the clusters of the presumes (#7, 8, 9), but not in the cingulate cortex (#10) (uncorrected *p*-values 0.036, 0.017, 0.047, and 0.069, adjusted *p*-values 0.067, 0.67, 0.67, and 0.069, respectively). The result of correlation analysis for each cognitive domain is shown in the Supplementary Information. No relationship was observed in OHV between degrees and scores on cognitive assessments in any of the clusters that were significantly different from YHV [mPFC, globus pallidus, or hippocampus [#1, 2, 12, 14], uncorrected *p* > 0.15].

**Figure 4:**
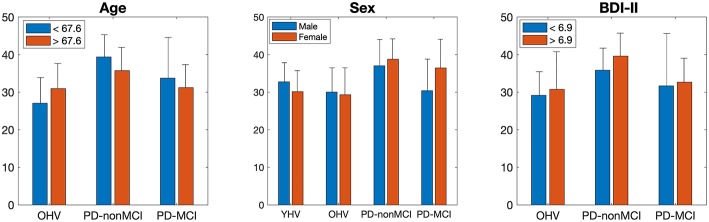
Mean of the averaged degree of the precuneus and cingulate cortex, in each subgroup of age, sex, and BDI-II. The bar indicates the S.D.

## Discussion

### Step 1

We investigated the connectivity differences among HV, PD-non-MCI and PD-MCI, applying BASC (36) and whole brain connectome with different resolutions (18). Our main observation was significant connectivity differences between PD-non-MCI and PD-MCI, without age covaried-out, across all the resolutions (Table 3). This result is in line with previous studies of PD patients with MCI (57–62). Here, the sensitivity was higher with lower resolution (<50), and lower but stable with higher resolution (>50). This is in agreement with studies in different patient groups using the same methodology (18, 63). The group difference was most prominent in connectivity with the medial cortex, including the primary motor cortex (Figure S3). Thus, considering the fact that the main symptom of the PD is motor dysfunction, the cognitive impairment might show pathophysiological overlap with motor dysfunction in PD patients, i.e., the connectivity impairment could potentially be attributed to the impairment in midbrain dopamine projections to the striatum. Interestingly, the difference between the OHV vs. PD-MCI was weaker, and only emerged when including age as a covariate (Table 3). In addition, in these comparisons the sensitivity was not higher with lower resolution (<50), which is atypical with this method (18, 63). Thus, we speculated that the reduction of the connectivity does not occur linearly as disease progresses. Instead, connectivity might be increased in some regions in PD-non-MCI patients likely reflecting a compensatory mechanism. In fact, increased and decreased overall connectivity has been reported in non-MCI and MCI PD patients compared to HV (57, 60).

### Step 2

Applying graph theory on the same rs-fMRI data in the first step combined with rs-fMRI data from YHV, we investigated the age, pathological condition, and cognition effects separately, on the hub regions of the brain. The results indicated that (1) decreased hub function mainly in the mPFC in the OHV compared with the YHV, (2) increased hub function in the posterior medial structures (presumes and cingulate cortex) in the PD-non-MCI compared to PD-MCI, with and without age covariate out, and (3) positive correlation between the hub function in the medial structure and cognitive level in all PD patients.

#### Compatibility With Step 1

Based on the results of the first step, we hypothesized a possible increased connectivity in the PD-non-MCI patients. With a different approach here, we found that the degree of the medial structures (cingulate cortex and the precuneus) was increased in the PD-non-MCI group compared to the OHV, in agreement with our hypothesis. In step 1 analyses, when age was included as a covariate, no significant difference was observed for the group comparison between PD-non-MCI and PD-MCI. However, here we observed that the increased degrees of the medial structure survived when accounting for the effect of age. By also comparing OHV and YHV, we confirmed that the increase of the posterior medial structure was not associated with healthy aging, but rather occurred exclusively in the PD-non-MCI patients. The two approaches to the data broadly support each other.

#### Medial Prefrontal Cortex (mPFC)

We observed a high degree and betweenness centrality in the mPFC, corresponding to the pre-supplementary motor area (pre-SMA) and supplementary motor area (SMA), in the YHV. This is in agreement with previous studies of functional and anatomical connectivity, indicating these regions as hub connectors (25, 28, 64). The mPFC is considered to be an important region in learning associations between events and in linking adaptive responses (65). The higher degree and betweenness centrality in YHV could support this cognitive function. Both network indices were decreased in the OHV compared to the YHV in these regions. This is in agreement with previous studies that have found that reduced connectivity of these regions is associated with cognitive decline in aging (27, 28). However, in the present data, no significant correlation was observed between the degree or betweenness centrality of the mPFC and the average cognitive Z-scores in the OHV. The discrepancy might be due to methodological differences, such as using different imaging parameters and analyses, or by participants' demography. In particular, we carefully excluded participants with any MCI in this group, which restricted the range of cognitive scores and could have resulted in selection a sample of participants that are making optimal use of an existing neural network.

No significant difference was observed between the OHV and PD-non-MCI groups in these regions. Thus, loss of connectivity in these regions may be primarily attributed to age rather than pathology.

#### Posterior Medial Structures (Cingulate Cortex and Precuneus)

In the PD-non-MCI patients, degrees were significantly increased in the cingulate cortex and the precuneus relative to both OHV and PD-MCI, and this effect was not accounted for by age. Of note, additional direct comparison indicated that compared with YHV, the PD-non-MCI patients showed significantly increased degrees in the precuneus (#7, 9), and the cingulate cortex (#10) (adjusted *p*-values 0.048, 0.023, and 0.054, respectively). Overall, this pattern suggests that PD-non-MCI patients recruit more the posterior medial structures and the functional regions they are associated with, but that this may be lost in PD-MCI patients. Moreover, the degree of these regions in all PD patients was positively correlated with the mean Z-score of the cognitive assessment (Figure 3). This is in agreement with our previous study, showing recruitment of the precuneus in PD patients during a set-shifting task (35). This supports the possibility that increased network activity in posterior medial structures might reflect compensatory mechanisms that protect against cognitive impairment in PD patients.

When each cognitive domain was considered, the degrees in the cingulate gyrus were correlated with the Z-scores of the attention domain, and the degrees in the precuneus were correlated with the Z-scores of the language domain. Moreover, the degrees in these clusters were marginally correlated with the Z-scores of the executive and memory domains (see Supplementary Information). Thus, the important function of the posterior medial structure in cognition could be a general function for cognition, i.e., hub function, plausibly to connect brain regions supporting specific cognitive functions, rather than being involved in specific cognitive function itself.

### Aging, a Risk Factor for Cognitive Decline in PD Patients

As described above, we observed reduced hub function in the mPFC in OHV, but not in the PD-non-MCI. Instead, we observed increased hub function in the posterior medial structures in this latter group. The mPFC is a key region associated with cognitive decline observed in aging (27, 66). Thus, for PD patients, the mPFC could be an important hub region for good cognitive performance. However, the mPFC is one of the important output of the cortico-basal ganglia thalamocortical loops (29, 67), and the hub function for connecting the basal-ganglia and the motor area. This function seems to be impaired in PD patients (33). Moreover, pathological change in the mPFC were observed in PD patients (68), and decreased dopaminergic function in the mPFC is associated with dementia in PD patients (69). Thus, depending on its integrity, the mPFC may not be able to act as the main hub for cognition in PD patients. Interestingly, a front-cingulo-parietal module (including the frontal area, basal ganglia, and precuneus) acts as a connector in young people, but this module is divided into two modules (the fronto-striatal-thalamus and medial posterior) in older people (28). PD-non-MCI patients are likely to recruit only one part of the divided two modules.

In the PD-non-MCI patients, the degree in the posterior medial structure was increased compared to the OHV. However, the betweenness centrality was not significantly higher in PD-non-MCI compared with OHV, even before multiple comparison correction (*p* > 0.15). The betweenness centrality is the total number of all shortest paths linking to the given node (52). Thus, the recruited connectivity which could increase degrees, may not represent fully optimized information transfer in the brain.

Additionally, we investigated the impacts of age, sex, BDI-II, one by one, on the degrees in the posterior medial structure, by dividing each group into two subgroups. The results indicated main effect of the group (PD-non-MCI vs. PD-MCI), but not the effect of the subgroup, or interaction of the group × subgroup. However, in the younger (< 67.6) and in the female participants, the averaged degree was strongly higher in PD-non-MCI compared with OHV (Table 4), and a marginal interaction was observed between the two groups (OHV vs. PD-non-MCI) and the age (< 67.6 vs. >67.6). Studies indicate, in PD patients, risk factors of cognitive impairment are age, male gender, depression, education, and severity of motor symptoms (7, 8). Our results indicate that younger and female PD patients may be able to recruit the posterior medial structure as hub more efficiently, compared with the older and male PD patients, giving a positive impact of cognition. However, given the small number of the participants for each subgroup, further studies are required to confirm this finding.

#### Basal Ganglia and Hippocampus

We observed the basal ganglia and the hippocampus as non-hub regions, in agreement with previous studies (28).

In the basal ganglia (globus pallidus), the degree was increased in OHV, compared with YHV. In older people, some parts of the basal ganglia are likely to increase the importance of connector function in the brain (70, 71), supporting their motor and cognitive performance (71, 72). However, the basal ganglia are the main pathological target of the PD. Therefore, cognitive benefit possibly relating to increased degree in the basal ganglia would be limited to PD patients. Nevertheless, no difference was observed in the degrees between OHV vs. PD-non-MCI. The globus pallidus shows increased connectivity with the medial temporal region and the posterior medial structure in older people, compared to younger people, but has decreased connectivity with the somatomotor cortex (70). Thus, the observation may reflect the further increased connectivity with the posterior medial structure region in PD patients, rather than involvement in the traditional cortico-basal-ganglia-thalamocortical loops (29). More studies are required to confirm this observation.

In the hippocampus, degrees was decreased in OHV compared with YHV, in line with previous studies (73), and no difference was between OHV vs. PD-non-MCI. Although the hippocampal function in the PD patients plays an important role for supporting cognition (35, 39, 56), pathological change of dopaminergic system is also observed in the hippocampus in PD patients (68), associating with cognitive impairment in advanced stages of PD (55).

The increased and decreased degrees in the globus pallidus and the hippocampus between YHV vs. OHV were only observed without multiple comparisons (Figure 2). The impacts of the connectivity in these regions on cognition in PD patients while important, might be less crucial than connectivity in the medial structures.

## Conclusion

Using rs-fMRI data with two different analyses, we investigated the effects of age, pathology, and cognitve impairment on brain connectivity in PD patients. Cluster connectivity and graph theory analysis provided distinct but convergent information about these processes. The comparison of the connectivity strength indicated the reduction of the multiple connectivities in PD-MCI patients compared to PD-non-MCI. Results were not strongly influenced by cluster number and location, but differences were reduced when age was included as a covariate. Using a graph-theory approach, we observed (1) decreased hub function mainly in the mPFC in OHV compared with the YHV, (2) increased hub function in the posterior medial structure (precuneus and the cingulate cortex) in PD-non-MCI patients, and (3) positive correlation between the hub function in the medial structure and cognitive function in all PD patients. Because of our small sample size, our interpretations should be taken with caution. Nevertheless, based on our results together with those of previous studies, we propose that a combination of hub modifications affect cognition in PD including (1) age-related reduction of hub function in the mPFC, and (2) recruitment availability of the posterior medial structure possibly to compensate for damaged basal ganglia function in PD-non-MCI.

## Ethics Statement

This study was carried out in accordance with the recommendations of the tri-council of Canada, Research Ethics Committee of the Regroupement Neuroimagerie Québec with written informed consent from all subjects. All subjects gave written informed consent in accordance with the Declarations of Helsinki. The protocol was approved by the Research Ethics Committee of the Regroupement Neuroimagerie Québec.

## Author Contributions

AN-S contributed to study design (second part), data acquisition, analyses, and writing. PB contributed to study design, methodological support, and analyses. AH, SJ, BM-C, and JL contributed to data acquisition A-LF and CD contributed to clinical feedback. KS and CB contributed to writing. OM contributed to study design, analyses, and writing.

### Conflict of Interest Statement

The authors declare that the research was conducted in the absence of any commercial or financial relationships that could be construed as a potential conflict of interest.
