# Supplementary material for: Why Is Aging a Risk Factor for Cognitive Impairment in Parkinson's Disease?—A Resting State fMRI Study
Source: Front Neurol. 2019 Mar 22;10:267. doi: 10.3389/fneur.2019.00267 (PMC6438889; doi:10.3389/fneur.2019.00267)
Supplement: Supplementary file 1 [file Data_Sheet_1.docx]

## **Supplementary information for**

## **“Why is aging a risk factor for cognitive impairment in Parkinson’s disease? – A resting state fMRI study – “**

Atsuko Nagano-Saito MD, PhD^,1,2^ Pierre Bellec PhD,^1,3^ Alexandru Hanganu MD, PhD,^1,4,5^ Stevan Jobert MSc,^1^ Béatriz Mejia-Constain PhD, ^1^ Clotilde Degroot MSc, ^1,3^ Anne-Louise Lafontaine MD, MSc, ^6,7^ Jennifer I. Lissemore, ^2^ Kelly Smart, ^2^ Chawki Benkelfat, ^2^ Oury Monchi PhD, ^1,3,4,5,7^

1 Centre de Recherche, Institut Universitaire de Gériatrie de Montréal, QC, CA

2 Department of Neurology, Neurosurgery, and Psychiatry, McGill University, Montreal, QC, CA

3 Université de Montréal, Montréal, CA, QC, CA

4 Cumming School of Medicine, Hotchkiss Brain Institute, Calgary, AB, CA

5 Department of Clinical Neurosciences and Department of Radiology, University of Calgary, Calgary, AB, CA

6 Movement Disorders Unit, McGill University Health Center, Montréal, QC, CA

7 Montreal Neurological Hospital, Department of Neurology, Montreal, QC, CA

8 Centre Hospitalier de l’Université de Montréal, Montréal, QC, CA

**Methods**

**Step2. Graph theory methodology**

Figure S1. ‘Cost efficiency’ and omega.

**
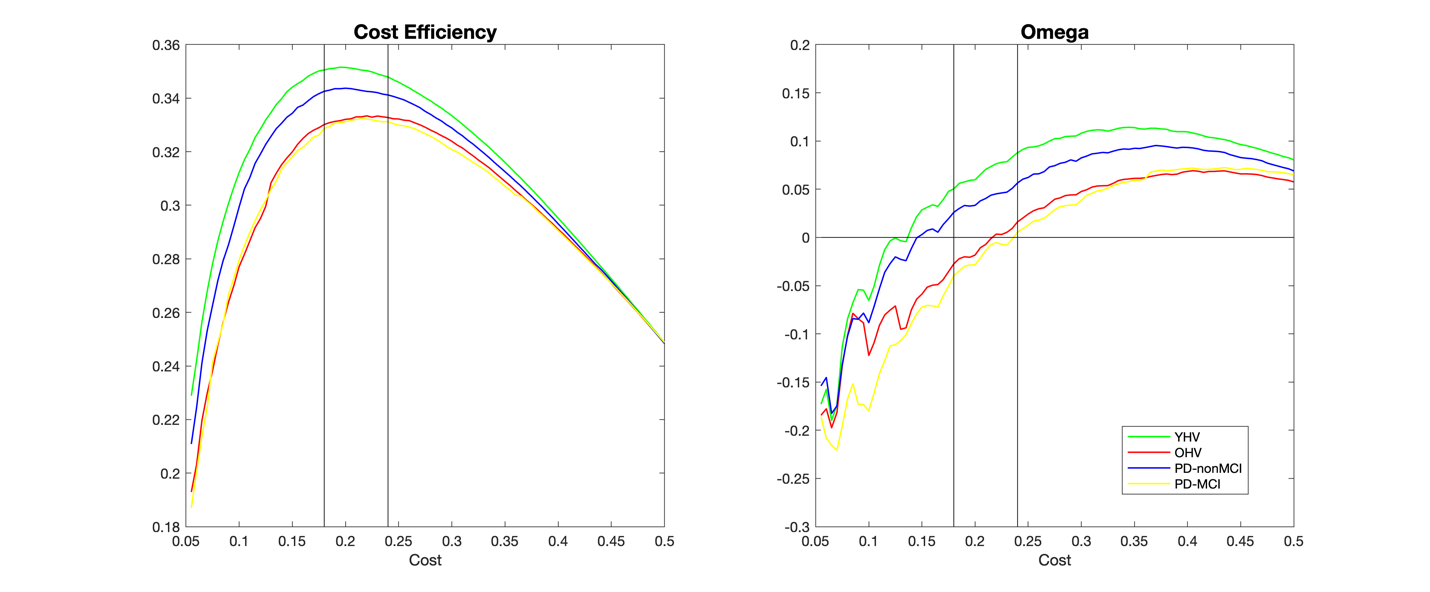
**

**Results**

**Cognitive assessment**

Figure S2. Mean Z scores of each domain.


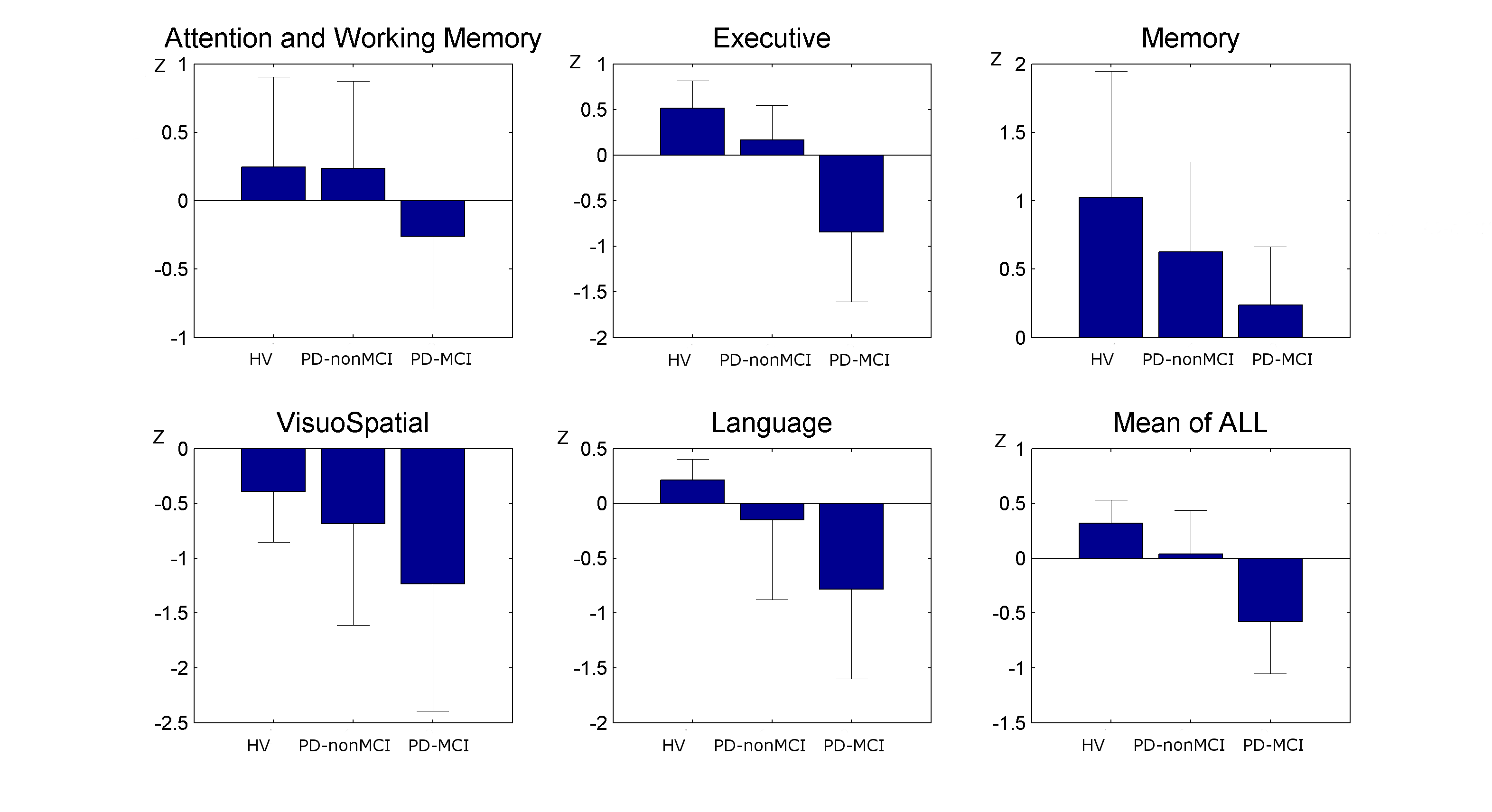


**Image Analysis of the step 1 in the manuscript**

Figure S3. Discovery percentage maps. Map of the percentage of connections associated with a given cluster and identified as significant by the statistical comparison between groups, and correlation with the cognitive scores (qFDR ≤ 0.05).


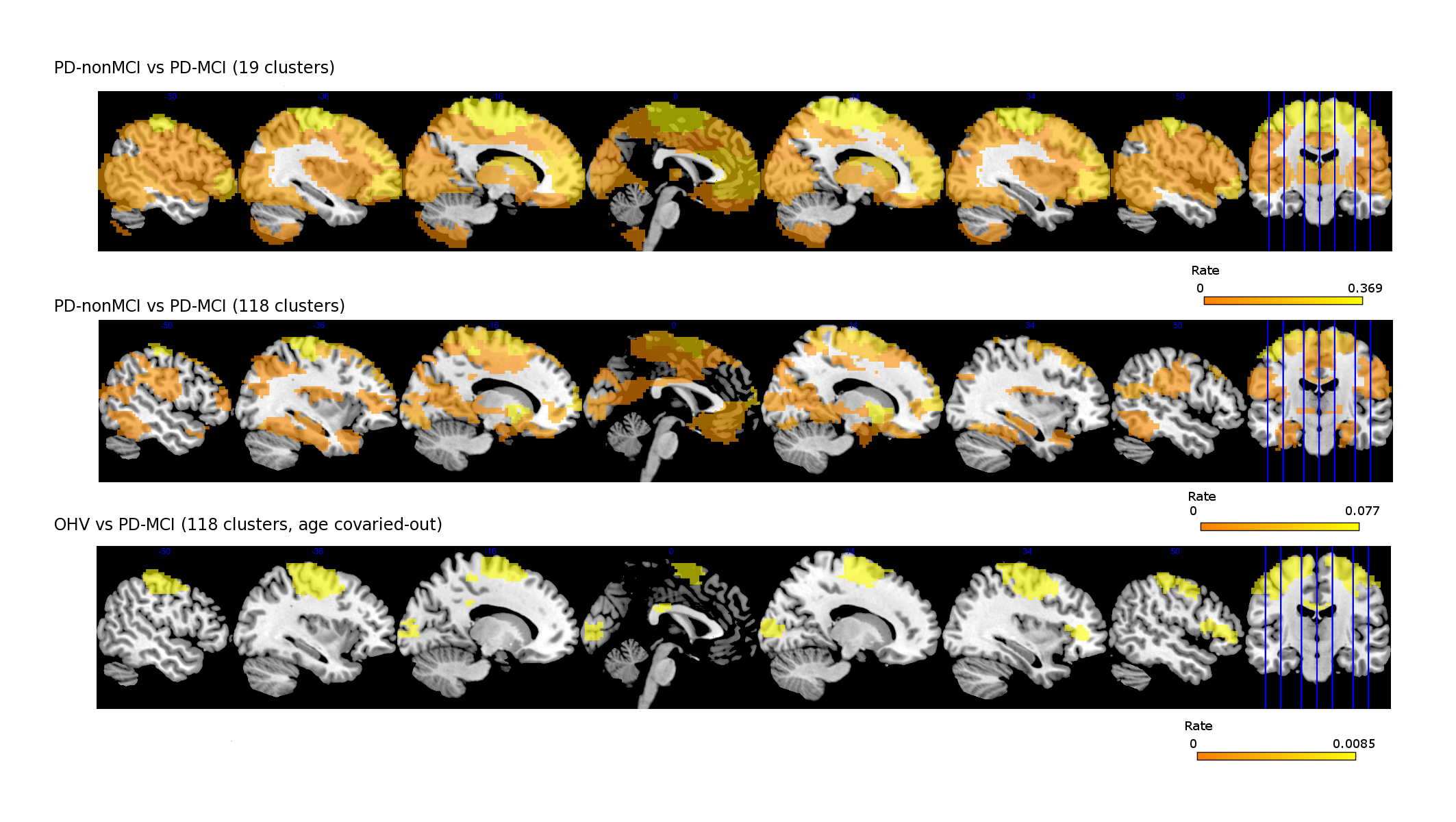


When comparing PD-nonMCI with PD-MCI (without age covaried-out), with resolution 19, group difference was observed almost all the brain except the temporal area and the upper cerebellum, indicating decreased connectivity in the PD-MCI (Figure S3, top). Discovery rate were prominent in the medial part of the cortex corresponding to the motor area. With the resolution 118, similar, but weaker group difference was observed (Figure S3, middle). When comparing HV with PD-MCI (with age covaried-out), with resolution 19, no difference was observed. With resolution 118, significant differences were observed in the medial frontal cortex, corresponding to the motor cortex, posterior cingulate cortex, right anterior prefrontal cortex, and occipital area (Figure S3, bottom).

**Detail results of ‘step 1. Connectivity difference between the PD-nonMCI and PD-MCI, and between the HV and PD-MCI’**

Additionally, here we present the group difference with respect to regional connectivity of specific clusters. In the Table S1, main clusters showing significant difference are summarized. (Note: the numbers are different from the main manuscript.)

**Table S1. Cluster number (#) and location.**


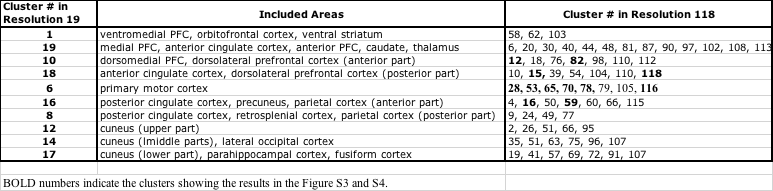


In the clusters located in the medial regions of the brain, for the PD-nonMCI vs. PD-MCI comparison (without age covaried out), with resolution of 19, most of the medial part of the brain (except #8, corresponding to the posterior cingulate cortex and retrosplenial cortex) showed significant group difference with respect to connectivity (PD-nonMCI > PD-MCI, without age covaried out). Overall, the spatially higher located clusters (#6 (the primary motor cortex), 10 (the dorsomedial prefrontal cortex, and the anterior part of the dorsolateral prefrontal cortex), 16 (the posterior cingulate cortex, the precuneus, and the parietal cortex), 18 (the anterior cingulate cortex, the posterior part of the dorsolateral prefrontal cortex) showed difference with the spatially lower areas, including the caudate nucleus, ventromedial cortex, insular cortex, occipital cortex, whereas the posterior and lower located clusters (#12 (the upper part of the cuneus), 14 (the middle part of the cuneus and the lateral occipital cortex), 17 (the lower part of the cuneus, the parahippocampal cortex, and the fusiform cortex), 19 (the medial prefrontal cortex, the anterior cingulate cortex, the anterior prefrontal cortex, the caudate, and the thalamus, 1) showed difference between the sensorimotor area, prefrontal cortex, and parietal cortex. Especially, the cluster #6, which included the primary motor area extending to the lateral part, showed most widely spread difference (Figure S3, top) over the brain including the striatum, thalamus, insular cortex, orbitofrontal cortex, and occipital area (Figure S4). The cluster #6 with resolution of 19 was divided into 8 clusters (#28, 78 70, 116, 65, 53, 105, and 79) of the resolution 118 (Table S1). The clusters #78, 28, 70, and 65 showed difference in striatum connectivity (Figure S5). The cluster #53, located in the left lateral motor cortex, showed difference with the caudate nucleus and the hippocampus, whereas the cluster #116, located in the right anterior lateral, and left posterior lateral motor cortex, showed difference with the occipital and ventromedial prefrontal cortex (Figure S5). Neither cluster #105 nor #79, located in the right posterior lateral motor cortex showed difference. Between HV vs PD-MCI (age covaried out), significant differences (HV > PD-MCI) were observed between the #78 and the occipital cortex, and between the splenium of the corpus callosum (cluster #1, located under cluster #16) and the right anterior frontal cortex.

Figure S4. Group difference between the PD-nonMCI and PD-MCI, with medially located clusters (qFDR < 0.05). The cluster number is 19.


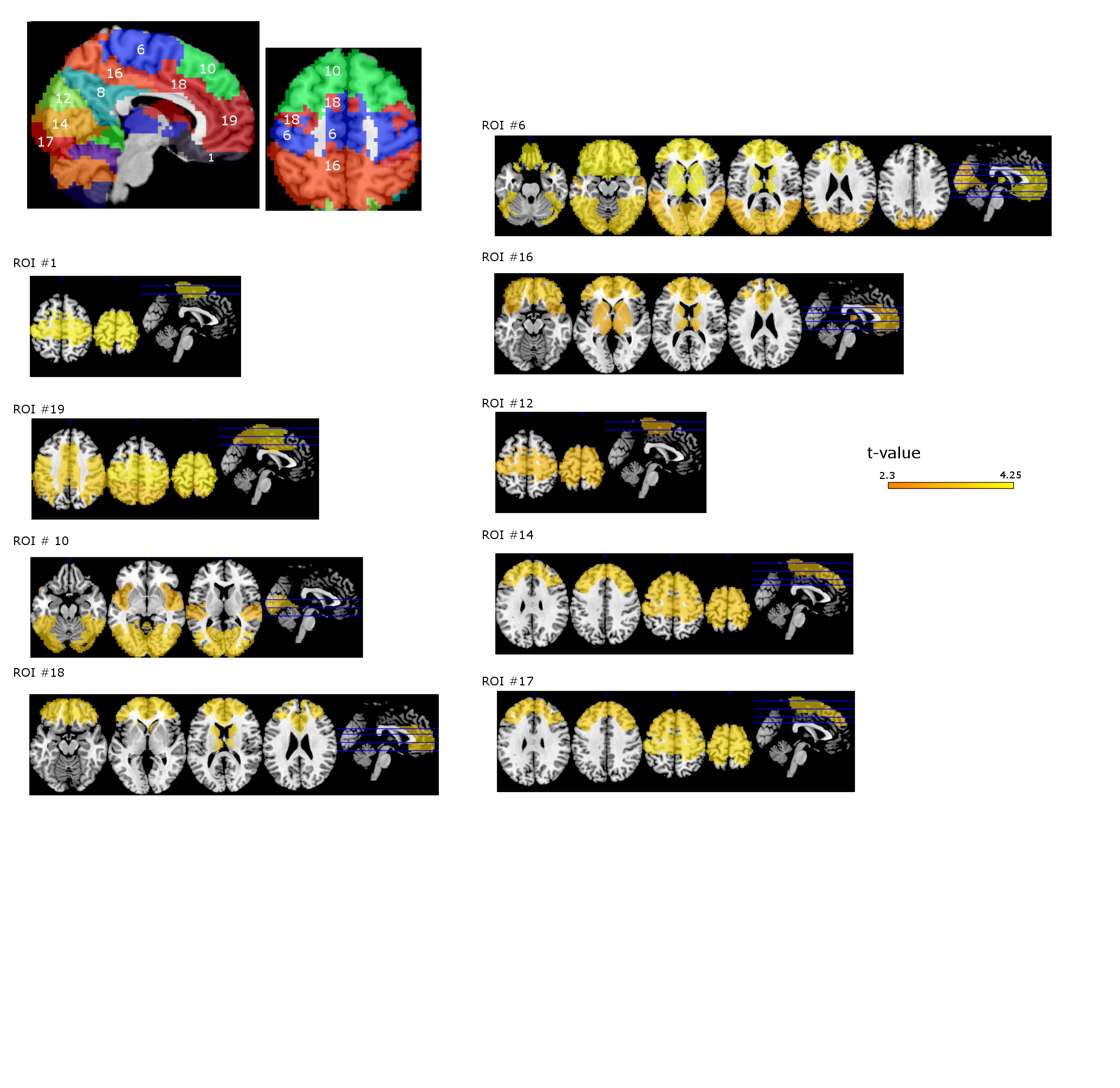


Figure S5. Group difference between the PD-nonMCI and PD-MCI, with medially located clusters (qFDR < 0.05). The cluster number is 118.


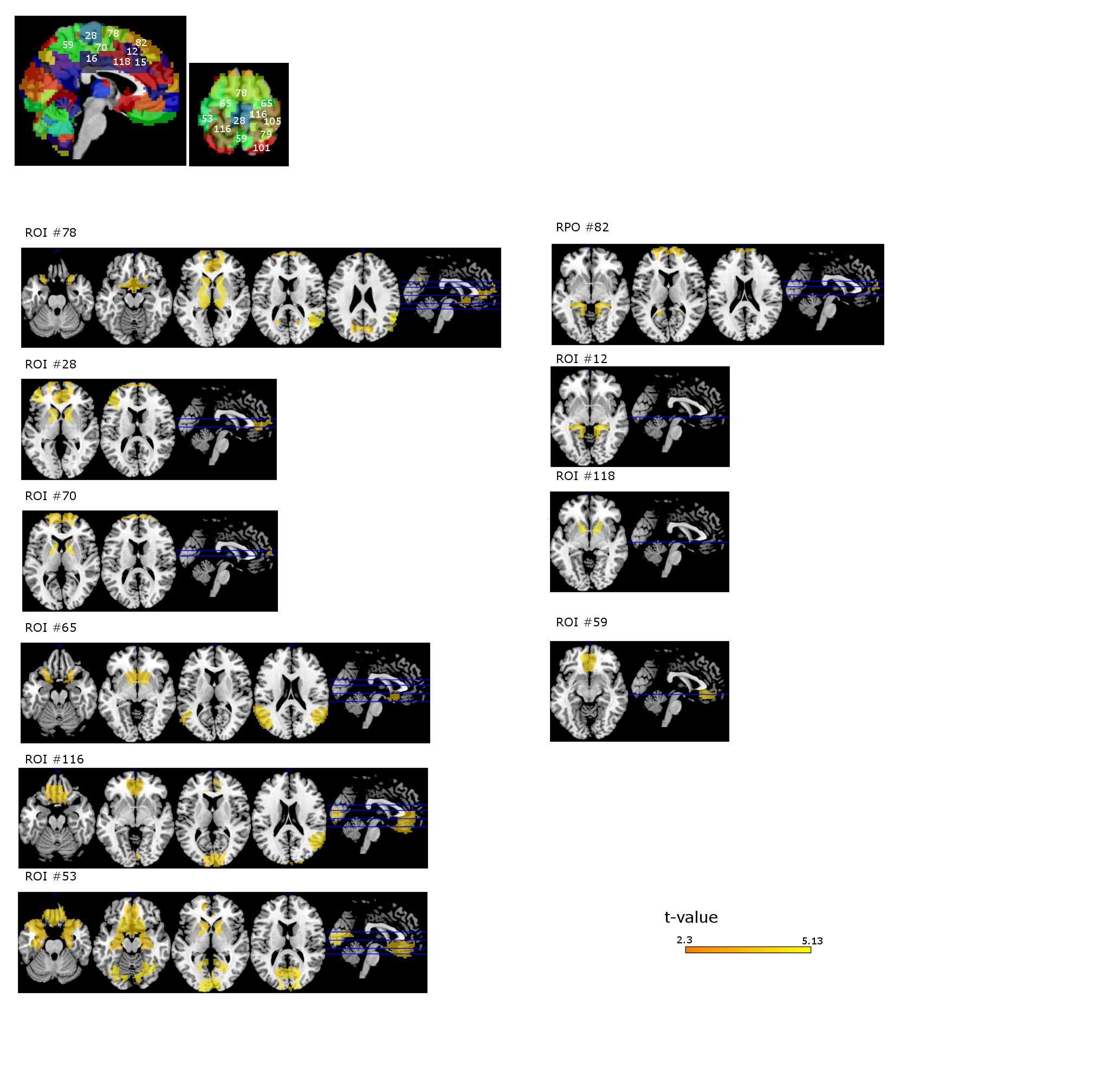


**Detail results of ‘step 2. Correlation between degrees and cognitive function’ considering each cognitive domain**

When considering Z-scores of each cognitive domain, without multiple comparison, the degrees in the cingulate gyrus (#10) was correlated with the Z-scores of the attention domain (r = 0.40; uncorrected p-value = 0.017), and in the precuneus (#7, 8) with the Z-scores of the language domain (r = 0.43 and 0.40; uncorrected p-value = 0.0096 and 0.018). When age was included as a covariate, all the correlations survived as significance (r = 0.38, 0.41, 0.39; uncorrected p-value = 0.028, 0.015, and 0.022, respectively). Besides, marginally significant correlation (uncorrected p < 0.1) were observed in the precuneus and the cingulate gyrus (#9, 10) with the executive domain, in the precuneus (#8) with the memory, and in the precuneus and the cingulate gyrus (#9, 10) with the language domain. All the correlation ration and p-value, without multiple comparison are shown in the Table S2 (without age as a covariate) and S3 (with age as a covariate).

**Table S2. Correlation between Z-scores of each cognitive domain and degrees in the precuneus and the cingulate cortex, without age as a covariate.**

| **cluster** | **correlation** | **Attention** | **Execution** | **Memory** | **Visuospacial** | **Language** | **Mean** |
| --- | --- | --- | --- | --- | --- | --- | --- |
| **#7** | **r** | 0.260 | 0.181 | 0.248 | 0.276 | **0.432** | **0.416** |
|  | **p-value** | 0.131 | 0.299 | 0.152 | 0.108 | **0.010** | **0.013** |
| **#8** | **r** | 0.182 | 0.266 | *0.285* | 0.267 | **0.398** | **0.416** |
|  | **p-value** | 0.295 | 0.122 | *0.097* | 0.120 | **0.018** | **0.013** |
| **#9** | **r** | 0.269 | *0.317* | 0.215 | 0.236 | *0.314* | **0.397** |
|  | **p-value** | 0.119 | *0.064* | 0.214 | 0.173 | *0.067* | **0.018** |
| **#10** | **r** | **0.401** | *0.321* | 0.182 | 0.041 | *0.318* | **0.346** |
|  | **p-value** | ***0.017*** | *0.060* | *0.295* | *0.814* | *0.062* | ***0.042*** |

**Table S3. Correlation between Z-scores of each cognitive domain and degrees in the precuneus and the cingulate cortex, with age as a covariate.**

| **cluster** | **correlation** | **Attention** | **Execution** | **Memory** | **Visuospacial** | **Language** | **Mean** |
| --- | --- | --- | --- | --- | --- | --- | --- |
| **#7** | **r** | 0.080 | 0.066 | 0.278 | *0.313* | **0.414** | **0.361** |
|  | **p-value** | 0.653 | 0.712 | 0.111 | *0.071* | **0.015** | **0.036** |
| **#8** | **r** | 0.373 | 0.25 | *0.291* | 0.275 | **0.391** | **0.407** |
|  | **p-value** | 0.157 | 0.154 | *0.095* | 0.116 | **0.022** | **0.017** |
| **#9** | **r** | 0.102 | 0.226 | 0.242 | 0.268 | *0.288* | **0.343** |
|  | **p-value** | 0.567 | 0.198 | 0.168 | 0.125 | *0.098* | **0.047** |
| **#10** | **r** | **0.376** | 0.279 | 0.193 | 0.053 | *0.302* | *0.316* |
|  | **p-value** | **0.028** | 0.11 | 0.273 | 0.768 | *0.083* | *0.069* |

**References for cognitive tasks (Table 2)**

Burgess P, Shallice T. The Hayling and Brixton Tests. Test manual. Bury St, Edmunds: Thames Valley Test Company; 1997.

Golden CJ, Freshwater SM. The stroop color and word test: a manual for clinical and experimental uses. Wood Dale, IL: Stoelting Co.; 1998.

Hooper HE. The hooper visual organization test manual. Los Angles, CA: Western Psychological Services; 1958.

Joanette Y, Ska B, Cote´ H. Protocole MEC - Protocole Montre´ al d’E´ valuation de la Communication: Ortho E´ dition, France. 2004.

Kaplan E, Googlass H, Weintrab S. Boston naming test. Philadelphia: Lea and Febiger; 1983.

Nasreddine ZS, Phillips NA, Be´ dirian V, Charbonneau S, Whitehead V, Collin I, et al. The Montreal Cognitive Assessment, MoCA: a brief screening tool for mild cognitive impairment. J Am Geriatr Soc 2005; 53: 695–9.

Osterrieth PA. Filetest de copie d’une figure complex: contribution a l’etude de la perception et de la memoire. Arch de Psychologie 1944; 30: 286–356.

Reitan RM, Wolfson D. The halstead-reitan neuropsychological test battery. Tucson: Neuropsychology Press; 1985.

Schmidt M. Rey auditory verbal learning test (RAVLT). Psychological Assessments. California: WPS publishing; 1996.

Wechsler D. Wechsler’s memory scale (WMS-III). New York: Psychological Corporation; 1997.

Wechsler D. Abbreviated Scale of IntelligenceTM (WASITM). New York: Psychological Corporation; 1999.
